# Impact of in vitro driven expression signatures of CD133 stem cell marker and tumor stroma on clinical outcomes in gastric cancers

**DOI:** 10.1186/s12885-019-5332-y

**Published:** 2019-02-04

**Authors:** Tae-Min Kim, Yoon Ho Ko, Shin Jung Ha, Han Hong Lee

**Affiliations:** 1 Department of Medical Informatics, The Catholic University of Korea, Seoul, South Korea; 2 Cancer Research Institute, The Catholic University of Korea, Seoul, South Korea; 3 Division of Medical Oncology, Department of Internal Medicine, The Catholic University of Korea, Seoul, South Korea; 4 Division of Gastrointestinal Surgery, Department of Surgery, College of Medicine, The Catholic University of Korea, 222 Banpo-daero, Seocho-gu, Seoul, 06591 South Korea

**Keywords:** Gastric cancers, CD133, Cancer stem cells, Expression signature, Survival

## Abstract

**Background:**

The CD133 transmembrane protein is a well-recognized stem cell marker that has been used to isolate putative cancer stem cell populations from gastric cancers (GCs). However, the molecular features or biomarkers underlying CD133 are largely unknown in GCs.

**Methods:**

We performed gene expression profiling of CD133+ and CD133- cells sorted by flow cytometry from three GC cell lines to identify the CD133 expression signatures of GC. The CD133 expression signatures were investigated across publicly available expression profiles of multiple tumor types including GC and also for their relationship with patient survival.

**Results:**

The CD133 signature genes defined as 177 upregulated genes and 129 downregulated genes in CD133+ cells compared to CD133- cells were enriched with genes involving the cell cycle and cytoskeleton, implying that cancer stem cells with unlimited self-renewal play cancer-initiating roles. The CD133 expression signatures in GC expression profiles were positively correlated with those of brain tumors expressing CD133 and human embryonic stem cells, emphasizing the transcriptional similarities across stem cell-related expression signatures. We also found that these stem cell expression signatures were inversely correlated with those representing tumor infiltrating immune and stromal cells. Additionally, high CD133 expression signatures were found in intestinal subtypes and low tumor stage GCs as well as in those with microsatellite instabilities and high mutation burdens. As examined across 20 additional tumor types, both the expression signatures representing CD133 and stromal cells were unfavorable prognostic features; however, their impact were variable across tumor types.

**Conclusions:**

The transcriptional activities of CD133 and those of stromal cells representing the activity of stem cells and level of epithelial-to-mesenchymal transition, respectively, may be inversely correlated with each other across multiple tumor types including GC. This relationship may be a confounding factor and should therefore be considered when evaluating the clinical relevance of stem cell-related markers.

**Electronic supplementary material:**

The online version of this article (10.1186/s12885-019-5332-y) contains supplementary material, which is available to authorized users.

## Background

Gastric cancer (GC) is a major human malignancy, with a high worldwide incidence and high morbidity [1]. Although endoscopy-based screening has greatly reduced the mortality and morbidity associated with this disease in East Asia, GC still causes many cancer-related deaths worldwide. Because of a lack of clinically approved biomarkers for advanced GC, it has been difficult to achieve a more detailed understanding of the molecular mechanisms underlying the initiation and progression of this disease to identify new prognostic factors and improve available therapeutic modalities [2].

CD133 is a well-known cancer stem cell marker of GC and has been used to isolate and functionally characterize GC stem cells [3]. Cancer stem cells are rare, quiescent, small cell populations with characteristic features of stem cells, such as unlimited self-renewal and lineage plasticity [4]. Immunohistochemistry (IHC)-based quantification of expressed CD133 protein levels has been proposed as a GC prognostic marker and CD133 positivity indicates poor prognosis as well as chemoresistance and disease progression of GC [5–12]. However, those reports have not considered the known heterogeneity issues of GCs [13]. It is important to understand the molecular mechanisms and underlying biology of CD133-associated cancer stem cells in a milieu of heterogeneous, non-tumor cells such as tumor-infiltrating stromal cells for a proper evaluation of this prognostic marker.

Instead of a single marker gene, the coordinated behavior of multiple genes involving shared molecular functions or cellular conditions has been sensitive and robust in identifying molecular functions in genomic profiles [14] or in predicting clinical outcomes [15]. For example, CD133 expression signatures (i.e., a summary score of the expression levels of CD133-related genes) have been previously identified in glioblastoma multiforme (GBM) and the investigators have proposed that CD133 expression signature can be used as a prognostic marker for GBM and other types of tumor [16].

Because of the clinical utility of CD133 as a biomarker of GC, it is important to identify the expression signatures associated with CD133 in GC and to determine their relationships with clinicopathological features. In this study, we performed microarray-based transcriptome analyses of CD133+ vs. CD133- cells obtained by cell sorting from three GC cell lines (KATO-III, SNU216 and SNU601). Using the CD133 expression signature, we examined transcriptional similarities with other stem cell-related signatures and investigated the relationship with the clinicopathological features of GCs. To evaluate the clinical impact of identified signatures, we also performed survival analysis for 21 tumor types including GCs.

## Methods

### Cell culture, flow cytometry, and fluorescence-activated cell sorting

Three GC cell lines (KATO-III, SNU216, and SNU601) were purchased from the Korean Cell Line Bank and maintained in RPMI1640 medium (Hyclone, Logan UT, USA) supplemented with 10% (*v*/v) calf serum (Hyclone) at 37 °C in a 5% (v/v) CO_2_ humidified atmosphere. The cells were harvested at 300×*g* for 5 min, incubated in cell-staining buffer containing phycoerythrin (PE)-labeled anti-CD133/1(AC133) antibody (1:10; Miltenyi Biotec, Bisley, UK) for 10 min in a dark refrigerator, and washed with 0.5% (*w*/*v*) bovine serum albumin in phosphate-buffered saline, pH 7.2, with 2 mM EDTA. An isotype-matched PE-labeled control antibody (Miltenyi Biotec) was used to label the samples and set gating levels. MoFlo XDP flow cytometry (Beckman Coulter, Brea, CA, USA) was also used to sort cell lines into CD133+ and CD133- populations. The data were analyzed using Summit software, version 5.2 (Beckman Coulter).

### Microarray analysis of the gene expression of CD133+ and CD133- gastric cancer cells

Total RNA was isolated from sorted cells using the Iso-RNA Lysis Reagent (Five Prime, South San Francisco, CA, USA) according to the manufacturer’s protocol. The extracted RNA, amplified and biotinylated using a TotalPrep RNA Amplification Kit (Illumina, San Diego, CA, USA), was quantitated using an Agilent 2100 Bioanalyzer. The Whole-Genome Expression Direct Hybridization Kit (Illumina) was used to hybridize 750 ng of cRNA from each sample to Human HT-12 v3 Expression BeadChips (Illumina) at 58 °C overnight. Unbound probe was removed by vigorous washing, and the BeadChip was scanned with a BeadArray reader (Illumina). The transcriptome profiles were quantile-normalized for the subsequent analysis. The microarray data were deposited in the GEO database (https://www.ncbi.nlm.nih.gov/geo; accession no. GSE112631).

### Clustering and gene set enrichment analyses

Hierarchical clustering was performed using 1000 genes with the highest variability or median absolute deviation (MAD). To identify differentially expressed genes, we used the signal-to-noise ratios (SNRs) of genes with SNRs > 1.0 and SNRs < − 1.0, which were used to identify the up- and downregulated genes, respectively, in CD133+ cells compared to CD133- cells (‘CD133-up’ and ‘CD133-down’, respectively). To construct a functional association map, we first performed Fisher’s exact tests for the up- and downregulated genes in CD133+ cells with Gene Ontology terms (MSigDB, c5 category; https://software.broadinstitute.org/gsea/msigdb/). Significantly enriched gene sets (*P* < 0.01) were collected and further examined for significant overlap of gene members across gene sets in a pairwise manner. Using Cytoscape, the gene sets and significant overlap (*P* < 1e-10; Fisher’s exact test) were presented as nodes and edges in a network topology [17].

### Quantitative reverse transcription polymerase chain reaction (qRT-PCR)

RNA isolation was performed using the RNeasey Mini Kit (Qiagen, Hilden, Germany) according to manufacturer’s protocol. cDNA was synthesized using amfiRivert cDNA Synthesis Platinum Master Mix (GenDEPOT, CA, USA). cDNA amplification for genes of interest was measured by amfiRivert qGreen Q-PCR master Mix (GenDEPOT) using a CFX96 Touch (Bio-Rad, CA, USA). Experiments were performed in triplicate for each set of primers.

### Signature activity and single-sample gene set enrichment analysis (ssGSEA)

Additional signatures were obtained from previously published reports. Expression signatures of genes that were differentially expressed in CD133+ and CD133- in primary GBM cells were then obtained [16]. Genes overexpressed in human embryonic stem cells (ESC) along with gene sets representing cell proliferation and the cell cycle were obtained elsewhere [18]. Signatures representing the tumor-infiltrating immune and stromal cells were obtained using the ESTIMATE package [19]. The expression signatures of collected gene sets including ‘CD133-up’ and ‘CD133-down’ were estimated using single sample gene set enrichment analysis (ssGSEA) [20] in large-scaled, RNA sequencing (RNA-seq)-based GC expression profiles from the Cancer Genome Atlas (TCGA) consortium [21]. Clinicopathological information from TCGA GC patients, including overall survival, were also obtained and used for correlative analyses with the CD133 expression signature. Additional dataset including microarray-based GC expression profiles and clinical information in an independent cohort [22], was also downloaded from GEO database (GSE62254). To test the impact of signature activity of CD133 expression and stromal cells on patient survival for multiple tumor types, we obtained the RNA-seq based gene expression level for additional 20 tumor types. Gene expression levels were downloaded (https://gdac.broadinstitute.org/) and signature scores were estimated using ssGSEA per tumor type. Those available for the overall patient survival were included in the multivariate Cox proportional hazard models. The TCGA annotation for 20 tumor types are BLCA (bladder urothelial carcinoma; *n* = 405), BRCA (breast invasive carcinoma; *n* = 1091), CESC (cervical and endocervical cancers; *n* = 304); COADREAD (colorectal adenocarcinoma; *n* = 375), ESCA (esophageal carcinoma; *n* = 184), GBM (*n* = 523), HNSC (head and neck squamous cell carcinoma; *n* = 518), KIRC (kidney renal clear cell carcinoma; *n* = 533), KIRP (kidney renal papillary cell carcinoma; *n* = 289), LGG (lower grade glioma; *n* = 514), LIHC (liver hepatocellular carcinoma; *n* = 370), LUAD (lung adenoma carcinoma; *n* = 506), LUSC (lung squamous cell carcinoma; *n* = 495), OV (ovarian serous cystadenocarcinoma; *n* = 302), PAAD (pancreatic adenocarcinoma; *n* = 178), PRAD (prostate adenocarcinoma; *n* = 497), SARC (sarcoma; *n* = 259), SKCM (skin cutaneous melanoma; *n* = 102), THCA (thyroid carcinoma; *n* = 501), and UCEC (uterine corpus endometrial carcinoma; *n* = 174).

### IHC-based CD133 classification of GC patients

Eighteen GC patients who underwent gastrectomy with combined lymph node dissection between January 2011 and December 2013 were enrolled in the study. This study was approved by the local Institutional Review Board (UC14SISI0137). The primary tumor specimens were snap-frozen. The frozen sections were stained with hematoxylin and eosin for histological examination for tumor purity (> 70%) by board-certified pathologists. The classifications of CD133+ and CD133- primary GC cases were based on IHC results from our previous report [7]. The expression-based signature scores of primary cases were also obtained by ssGSEA methods.

## Results

### CD133 signature genes in GCs

**Fig. 1:**
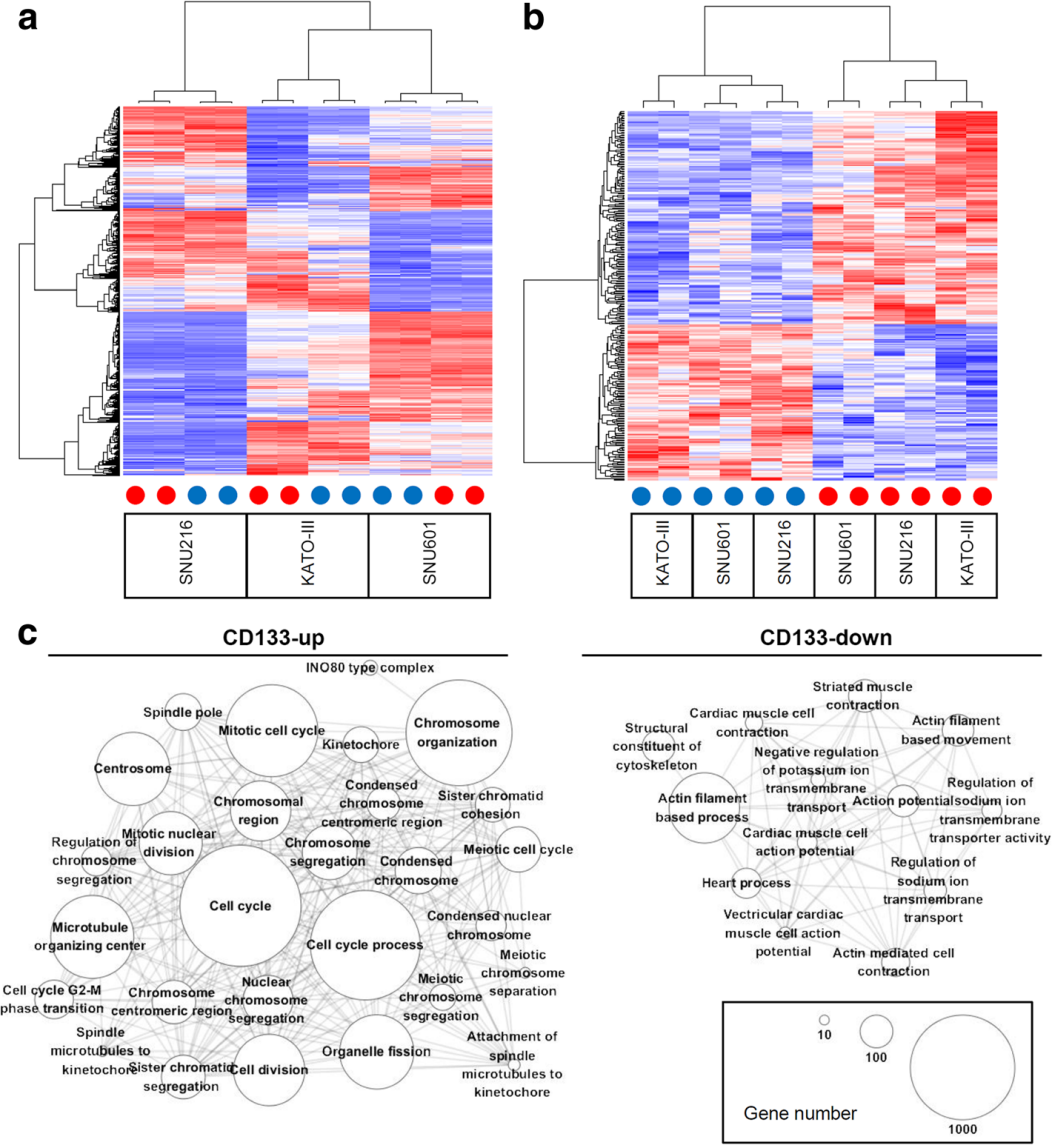
The CD133-associated expression signature of gastric cancer (GC) cell lines. **a** Hierarchical clustering of 1000 highly variable genes segregated in three GC cell lines (SNU216, KATO-III and SNU601). Red and blue dots represent CD133+ and CD133- cells, respectively. **b** The commonly over- and under-expressed genes in CD133+ as CD133 signatures segregated in the CD133+ and CD133- cells regardless of cell line. **c** A functional association map linked the Gene Ontology terms according to their significant overlap of gene members. Network topology demonstrated two main subnetworks representing the cell cycle-, cytoskeleton-, and transporter-related molecular functions enriched in CD133+ and CD133- GC cell lines, respectively. The node size corresponds to the number of genes in the function

To evaluate the gene expression associated with the CD133 stem cell marker in GCs, we performed gene expression profiling of three gastric cancer cell lines (KATO-III, SNU216, and SNU601). For each cell line, we separated CD133+ and CD133- cells using fluorescence-activated cell sorting and performed microarray-based gene expression profiling. The top 10% of CD133+ cells in terms of fluorescence intensity and the bottom 6% of CD133- cells were collected by flow cytometry. Fluorescence-activated cell sorting using CD133 antibody for gastric cancer cell lines is illustrated in Additional file 1: Figure S1. Hierarchical clustering segregated the three GC cell lines as well as the CD133+ and CD133- cells in each cell line, and these data were indicative of a substantial level of heterogeneity across cell lines examined (Fig. 1a). To consider heterogeneity between cell lines, we identified 177 genes commonly upregulated in CD133+ cells compared with CD133- cells (SNR > 1.0) across three cell lines and defined them as CD133 signatures (“CD133-up”). We also selected 129 genes as commonly downregulated genes in CD133+ cells compared with CD133- cells (“CD133-down” signature with SNR < − 1.0). A list of 20 up- and 20 down-regulated genes in CD133+ celllines compared to those of CD133- is shown in Table 1 with a full list of differentially expressed genes available in Additional file 2: Table S1. Using qRT-PCR, RNA expression level of the most differentially expressed genes in the list (CDC2 and ARG1) was evaluated. Primer sequences of two genes are shown in Additional file 2: Table S2. RNA expression of CDC2 gene was elevated in the three CD133+ gastric cancer cell lines. In terms of ARG1, RNA expression decreased in the CD133+ KATO-III and SNU216 cell lines (Additional file 1: Figure S2). Hierarchical clustering of 306 CD133 signature genes clearly segregated the CD133+ and CD133- cells across three cell lines (Fig. 1b). We also evaluated how many percentages of the sorted CD133+ and CD133- cells exhibit CD133+ and CD133- signatures using a gene expression-based deconvolution algorithm of CIBERSORT [23]. The algorithm revealed that CD133-up and CD133-down signatures are relatively enriched in the corresponding CD133+ and CD133- sorted cells (Additional file 1: Figure S3), suggesting that the identified signatures can be used as a measure for CD133 activity. The CD133 signature genes included *PROM1,* which encoded CD133 molecules (“CD133-up”), and *OVOL2,* whose encoded transcription factors have been previously implicated in epithelial differentiation and cancer progression (“CD133-down”) [24]. To further explore the molecular functions associated with CD133 signature genes, we performed functional enrichment analyses with Gene Ontology terms (MSigDB c5 category). The 29 and 15 functional categories substantially enriched (*P* < 0.01; Fisher’s exact test) with CD133-up and CD133-down signature genes, respectively, are listed in Additional file 2: Table S3. Figure 1c shows a functional association map where the nodes are functional categories enriched with CD133 signature genes, and the edges involve significant overlap (*P* < 1e-10; Fisher’s exact test) between them. In the network topology, CD133-up signature genes were largely enriched with genes of cell cycle-related functions, whereas CD133-down signature genes were implicated in the molecular functions of the cytoskeleton and transport.

**Table 1:** A list of 20 up- and 20 down-regulated genes in CD133+ gastric cancer celllines compared to CD133- celllines

Type	Symbol	SNR	RefSeq	Description
Up	CDC2	2.91	NM_001786.2	Cell division cycle 2, G1 to S and G2 to M
	SGOL1	2.38	NM_138484.2	Shugoshin-like 1 (S. pombe)
	TCHP	2.13	NM_032300.2	Trichoplein, keratin filament binding
	TOP2A	2.13	NM_001067.2	Topoisomerase (DNA) II alpha 170 kDa
	CENPK	1.85	NM_022145.3	Centromere protein K
	PAQR3	1.85	NM_001040202.1	Progestin and adipoQ receptor family member III
	HMMR	1.81	NM_012485.1	Hyaluronan-mediated motility receptor (RHAMM)
	LIN54	1.76	NM_194282.1	Lin-54 homolog (*C. elegans*)
	DCK	1.75	NM_000788.1	Deoxycytidine kinase
	ALG8	1.72	NM_024079.4	Asparagine-linked glycosylation 8, alpha-1,3-glucosyltransferase homolog (*S. cerevisiae*)
	SEPSECS	1.68	NM_016955.1	Sep (O-phosphoserine) tRNA:Sec (selenocysteine) tRNA synthase
	LOC642341	1.65	XM_930714.1	Hypothetical LOC642341
	BUB3	1.65	NM_004725.2	BUB3 budding uninhibited by benzimidazoles 3 homolog (yeast)
	KNTC1	1.56	NM_014708.3	Kinetochore associated 1
	MGC40489	1.55	XR_016048.1	Hypothetical protein MGC40489
	LOC100132861	1.54	XM_001716443.1	Hypothetical protein LOC100132861
	RFC5	1.53	NM_007370.3	Replication factor C (activator 1) 5, 36.5 kDa
	CR1L	1.50	NM_175710.1	Complement component (3b/4b) receptor 1-like
	PPT1	1.49	NM_000310.2	Palmitoyl-protein thioesterase 1 (ceroid-lipofuscinosis, neuronal 1, infantile)
	ACTL6A	1.49	NM_177989.2	Actin-like 6A
Down	ARG1	−1.76	NM_000045.2	Arginase, liver
	CPXM2	−1.59	NM_198148.1	Carboxypeptidase X (M14 family), member 2
	LOC440353	−1.58	NR_002603.1	Nuclear pore complex interacting protein pseudogene
	ALPK1	−1.56	NM_025144.2	Alpha-kinase 1
	KIAA0664	−1.51	NM_015229.3	KIAA0664
	MIR634	−1.51	NR_030364.1	MicroRNA 634
	LOC642446	−1.47	XM_001717781.1	Similar to hCG1795201
	FBXO7	−1.47	NM_012179.3	F-box protein 7
	LOC645812	−1.45	XM_928801.1	Similar to wingless-type MMTV integration site family, member 9B precursor
	POLR2C	−1.44	NM_032940.2	Polymerase (RNA) II (DNA directed) polypeptide C, 33 kDa
	LOC441124	−1.44	XM_499022.3	Hypothetical LOC441124
	FGL1	−1.44	NM_004467.3	Fibrinogen-like 1
	TMEM120A	−1.44	NM_031925.1	Transmembrane protein 120A
	LOC650909	−1.43	XM_939995.2	Similar to activating signal cointegrator 1 complex subunit 3-like 1
	LOC100129101	−1.41	XM_001721125.1	PHypothetical LOC100129101
	LOC728944	−1.41	XM_001128859.1	Similar to THAP domain-containing protein 4
	PARP12	−1.40	NM_022750.2	Poly (ADP-ribose) polymerase family, member 12
	SND1	−1.40	NM_014390.2	Staphylococcal nuclease and tudor domain containing 1
	CAMK2B	−1.40	NM_172081.1	Calcium/calmodulin-dependent protein kinase (CaM kinase) II beta
	LOC100132761	−1.40	XM_001716956.1	Hypothetical protein LOC100132761

Note: Gene symbols are shown with RefSeq annotations. Type indicates whether the gene is relatively up- or down-regulated in CD133+ celllines compared to CD133- celllines. SNR (signal-to-noise ratios) is the level of relative expression

### The CD133 expression signature of GC and other stem cell-related signatures

**Fig. 2:**
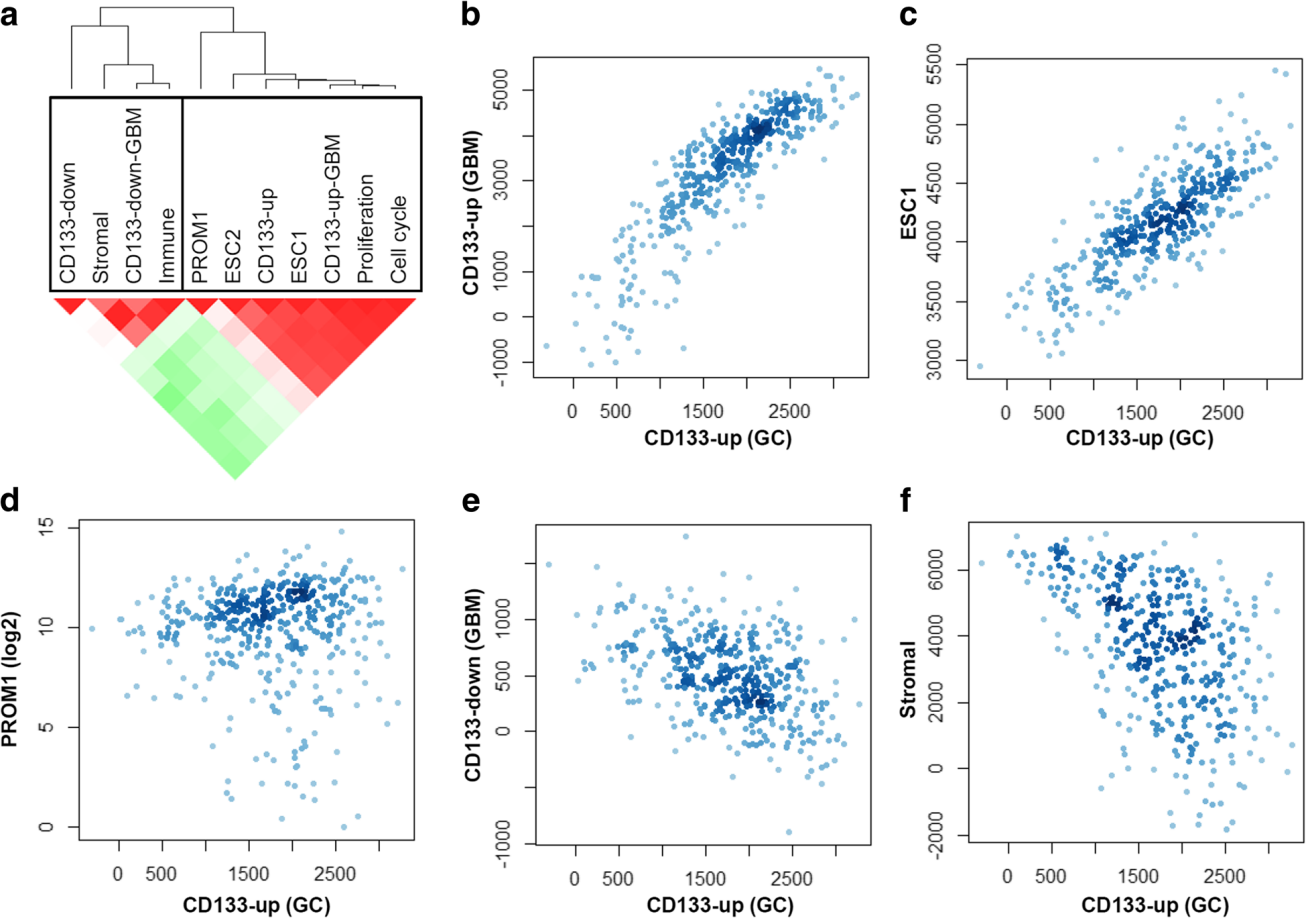
The relationships of stem cell-related signatures. **a** Hierarchical clustering of expression signature from multiple stem cell-related signatures and those of tumor-infiltrating immune and stromal cells segregated into two major clusters. The CD133 down [gastric cancer (GC) and glioblastoma multiforme (GBM)] clusters included stromal and immune signatures (left). CD133-up (GC and GBM) clusters included embryonic stem cell signatures (ESC1 and ESC2) and those representing cell proliferation and the cell cycle (right). A heat map shows the level of the Pearson’s correlation coefficient (red and green for higher and lower correlations, respectively) in a pairwise manner. **b**–**f** As noted, scatter plots show the distribution of TCGA GC samples according to the expression levels from stem cell-related signatures

To compare the CD133 expression signature of GC, the CD133 signature genes of primary GBM (“CD133-up-GBM” and “CD133-down-GBM” as these were up- and downregulated, respectively, in CD133+ GBM cells compared to CD133- cells) were obtained from previously published reports [16]. Signature genes representing those overexpressed in ESC were also obtained (“ESC1” and “ESC2”) along with signature genes annotated as cell proliferation and cell cycling (“Proliferation” and “Cell cycle”) [18]. To assess the contribution of tumor-infiltrating immune and stromal cells in the bulk tumor transcriptome, corresponding signature genes were obtained (“Immune” and “Stromal”, respectively) [19]. To evaluate the expression-based activity of stem cell-related signature genes, we performed ssGSEA on large-scaled GC gene expression profiles from the TCGA consortium (*n* = 425) [21]. The obtained enrichment scores, or the “‘expression signatures” in GCs, were examined for pairwise correlations. Using hierarchical clustering, we noted two clusters, each of which included the expression signatures of CD133-up and CD133-down (Fig. 2a). The CD133-up expression signatures of GC and GBM were correlated with each other as well as with two ESC expression signatures. This suggested that the CD133 expression signature levels were consistent across tumor types (GBM in vivo and GC in vitro) and were also correlated with those of human ESC with pluripotency. The concordance with expression signature levels representing cell proliferation and cell cycling also suggested that the observed stem cell-related expression signature levels were associated with a high level of proliferative potential and accelerated cell cycling. Of note, the CD133-down expression signatures of GC and GBM were correlated with each other and also with the expression signatures representing tumor-infiltrating immune and stromal cells. These estimates have been known to be inversely correlated with tumor purity and reflected the relative abundance of tumor-infiltrating immune and stromal cells [19]. Figure 2b–f shows the relationship between the expression signature levels using scatter plots. We also examined the overlap of signature genes. The highest overlap was observed between “Proliferation” (366 genes) and “Cell cycle” (653 genes) (104 genes overlapping). However, as the CD133-up (GC; 177 genes) signature genes showed that < 10% of genes overlapped with the other signatures, a mere gene overlap did not explain the observed correlation between the expression signatures.

### Clinicopathological features associated with CD133 expression signature in GC

**Fig. 3:**
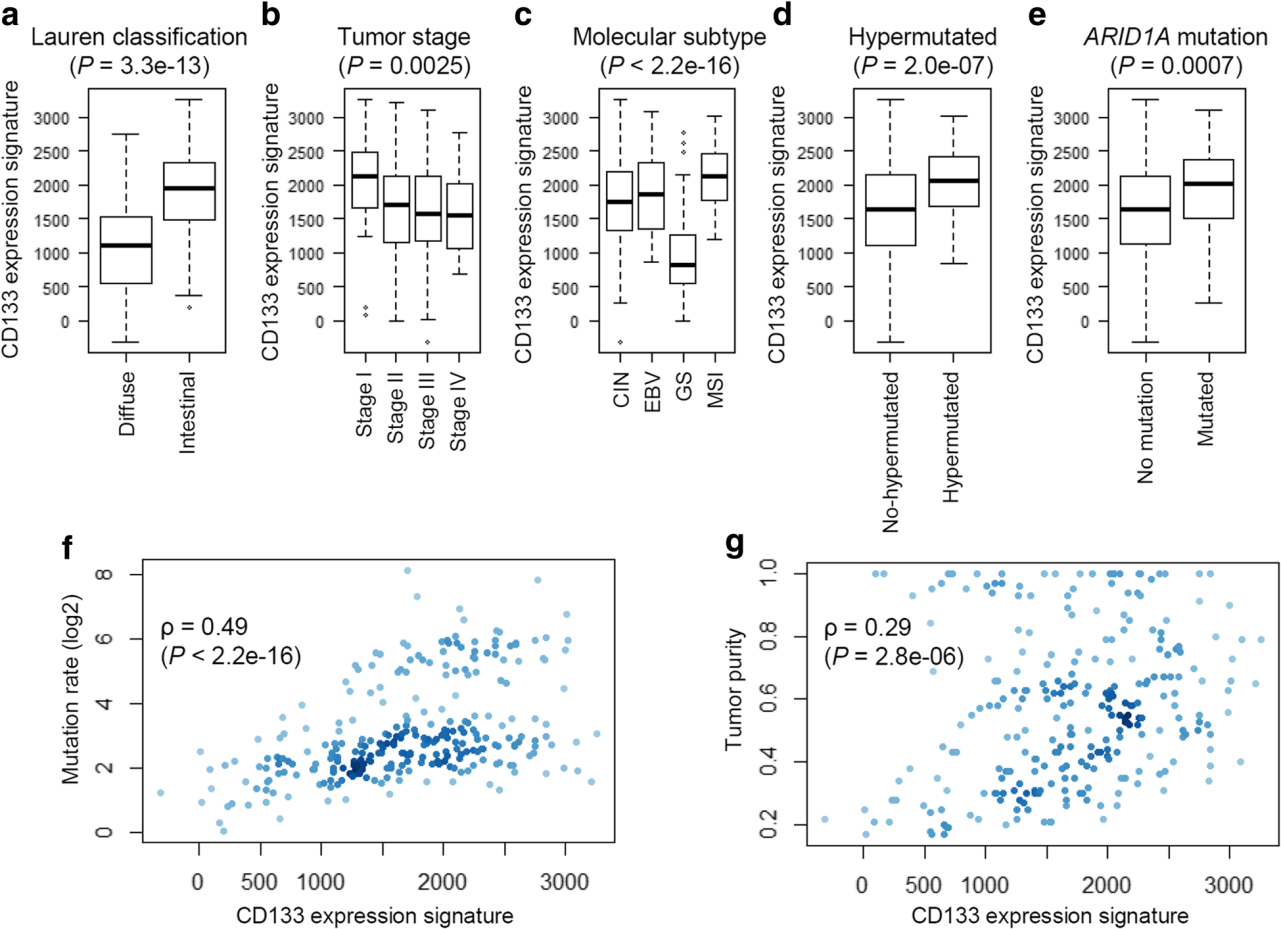
Clinicopathological features and CD133 expression signature levels. Among the clinicopathogical features examined, significant correlations with CD133 expression signature (*P* < 0.01) are shown. A complete list of the features examined and their statistical significance are available in Additional file 2: Table S3

We further evaluated the CD133 expression signature in terms of their correlation with or enrichment of the clinicopathological features of GC as available in the TCGA consortium (Fig. 3). Among the 33 clinicopathological features examined, we determined those significantly correlated (*P* < 0.01) with the CD133-up (GC) signature. Thirty-three clinicopathological features were listed with the statistical tests and the significance levels for their enrichments of or associations with CD133 expression signature levels (Additional file 2: Table S4). First, significantly higher CD133 expression signatures were noted for intestinal types compared to diffuse types (Fig. 3a; *P* = 3.3e-13) and also for stage I GC tumors (Fig. 3b; *P* = 0.0025). Among the four molecular taxa of GC previously proposed [21], high and low CD133 expression signatures were observed for GC with microsatellite instability (MSI) and genomically stable GC, respectively (Fig. 3c; *P* < 2.2e-16). The association of CD133 expression signature with MSI further showed that significantly higher CD133 expression signatures were observed in MSI-H (high) tumors compared to MSI-L (low) and MSS (microsatellite-stable) tumors as well as in tumors with DNA promoter methylation of *MLH1*, a well-known somatic alteration leading to MSI (Additional file 1: Figure S4). MSI-H GC tumors frequently show elevated mutation abundance and we also found high CD133 expression signatures in hypermutated GC tumors (Fig. 3d; *P* = 2.0e-07) with a significant correlation of the CD133 expression signatures and increased mutation burdens across individual GC tumors (Fig. 3f; *P* < 2.2e-16). Among the major mutations of GC, mutations of *TP53*, *PIK3CA*, *KRAS*, *ARID1A*, and *RHOA* were evaluated, whereas only *ARID1A* mutations were significantly associated with CD133 expression signature (*P* = 0.0007; Fig. 3e). Regarding tumor ploidy and purity, tumor purity was significantly associated with CD133 expression signature (*P* = 2.8e-06; Fig. 3g), which was consistent with the inverse correlation with the expression signatures representing tumor-infiltrating immune and stromal cells (Fig. 2a and f). We also investigated the relationship of CD133 signature levels with the selected clinicopathological features for an independent GC cohort [22]. We consistently observed that high CD133 signature levels for intestinal subtype, stage I tumors, MSI molecular subtype GC, and those negative for MLH1 IHC (Additional file 1: Figure S5).

### The relationship between CD133 expression signature levels and IHC

**Fig. 4:**
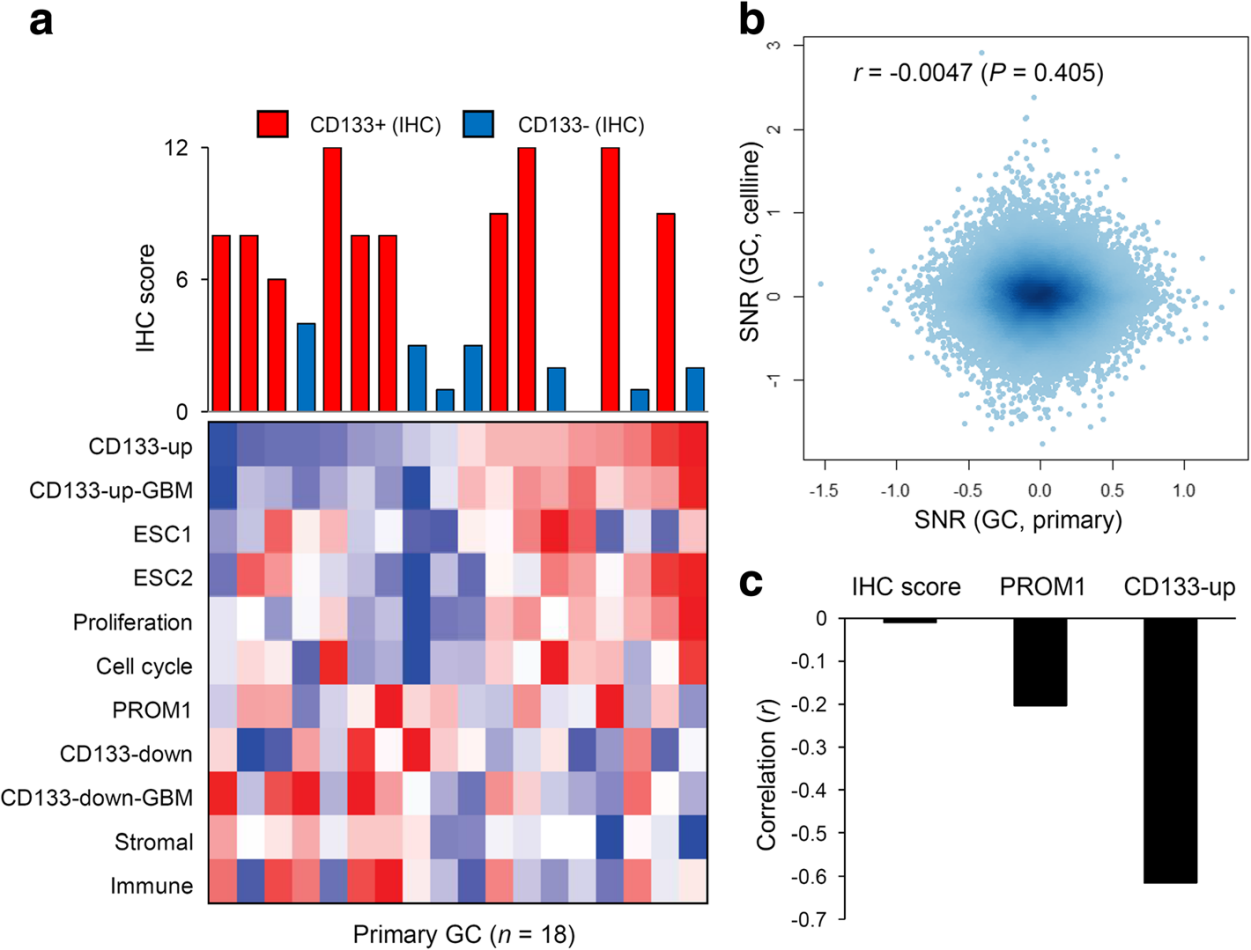
CD133 expression signature levels and immunohistochemistry (IHC)-based CD133 positivities in GC primary cases. **a** Eighteen GC primary cases were sorted in order of the expression signature level of CD133-up (the top row in the heat map). A barplot shows the level of IHC score (*y*-axis) with IHC-based CD133+ and CD133- primary cases (red and blue, respectively; upper panel). A heatmap shows the level of signature levels examined for 18 primary GC cases (below). **b** The signal-to-noise ratios (SNR) estimated from the cell lines (y-axis) and primary cases (x-axis) are shown in a scatter plot. No significant correlation was observed. **c** The correlation level with stromal signatures are shown for IHC score, PROM1 expression and CD133-up signature levels, respectively

The CD133 stem cell markers have been evaluated by IHC-based quantification of CD133 protein expression. To evaluate the CD133 expression signature levels in terms of the conventional IHC-based CD133+ and CD133- GC classifications, we conducted microarray-based gene expression profiling of 18 primary GC cancers (ten CD133+ and eight CD133- cases with the cutoff of IHC score of 6; Fig. 4). In the cohort, a correlation among the CD133 expression signature levels (Fig. 2a) was consistently observed, including an inverse correlation between the expression signature levels of CD133-up and stromal cells (Fig. 4a). Notably, there was no apparent relationship between the CD133 expression signature levels and IHC-based CD133 positivity. No significant difference in signature activity was observed between CD133+ and CD133- primary GC cases (*t*-test), including PROM1 expression. Additionally, the SNR (CD133+ vs. CD133-) estimated from the cell lines and primary GCs were not correlated with each other (*r* = − 0.0047; *P* = 0.405; Fig. 4b). In vitro-driven CD133 signature genes (177 and 129 genes with the cutoff of SNR 1.0 and − 1.0, respectively) do not overlap with similarly sized differentially expressed genes (154 and 144 genes with the cutoff of SNR 0.7 and − 0.7 for primary cases). Importantly, k-nearest neighbors (kNN)-based leave-one-out-cross-validation (LOOCV) tests based on gene expression achieved 100% accuracy in predicting CD133+ and CD133- annotations for the 3 cell lines used to construct the in vitro CD133 expression signatures. However, the prediction accuracy for the kNN-LOOCV test of 18 primary GC cases was less than expected by chance (< 50%).

When we calculate the correlation between the IHC score and PROM1 expression, we observed a substantial level of correlation (*r* = 0.447; *P* = 0.06). However, an inverse relationship was observed between the IHC score and CD133 signature levels (*r* = − 0.228; *P* = 0.362). We assume that this paradoxical relationship of IHC score with CD133 signature levels may be due to stromal contamination. IHC scores as evaluated by manual examination by pathologist adjusting for the non-tumor cell components such as stromal cell, are not correlated with stromal signatures level (*r* = − 0.01; Fig. 4c). But stromal signature showed a substantial level of inverse correlation with PROM1 expression and CD133 signature scores (*r* = − 0.203 and − 0.615, respectively; Fig. 4c). It is expected that epithelium-driven PROM1 expression and CD133 signature scores may be inversely correlated with the stromal signature scores and the stromal signature levels may be a confounding factor in the evaluation of the relationship between IHC score and CD133 signature scores as well as with PROM1 expression.

### The impact of CD133 expression signatures on survival

**Fig. 5:**
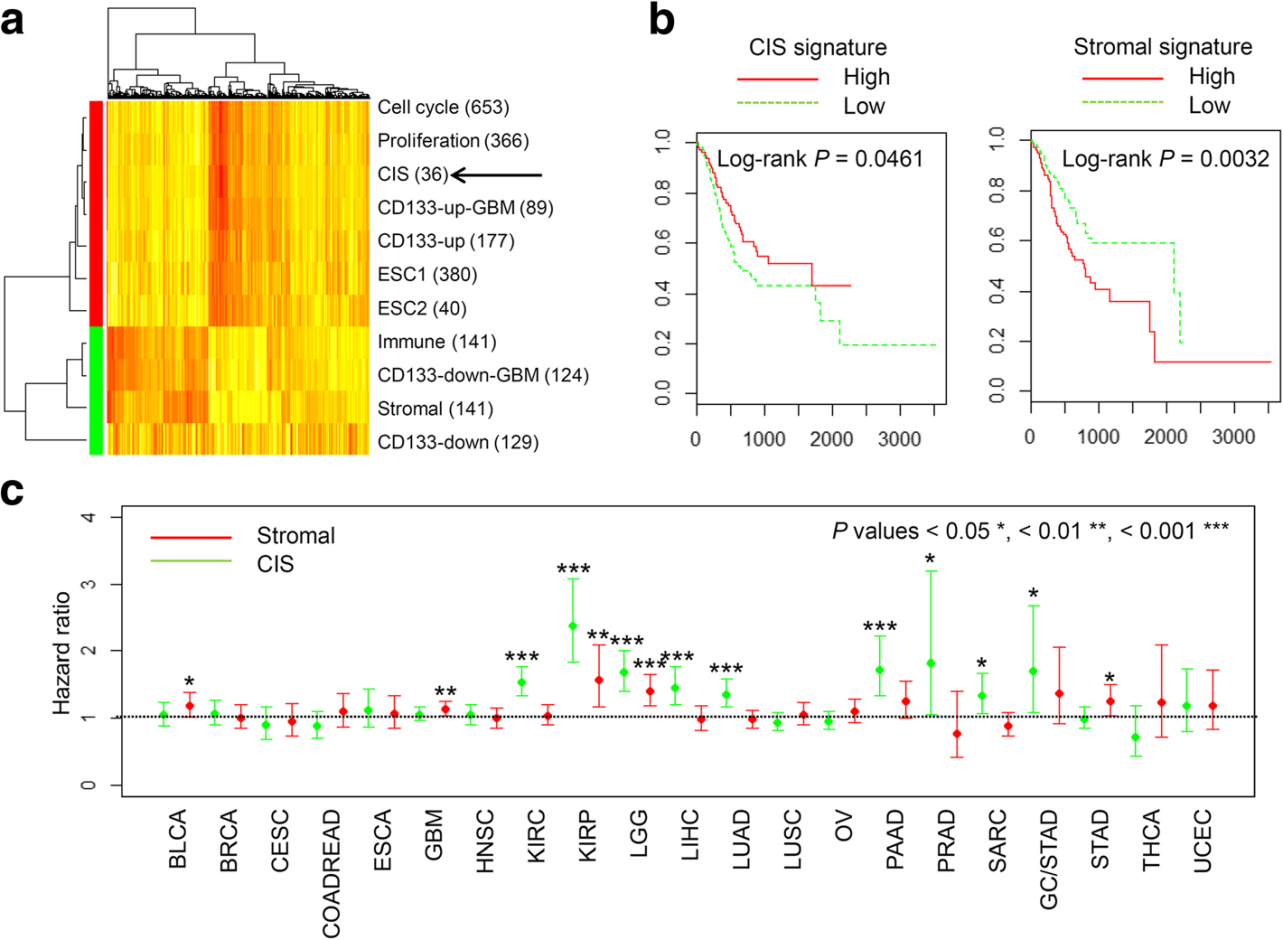
Patient survival with respect to the expression signature of CD133 and stromal cells. **a** A heatmap shows the relationship of the signature levels including CIS signature (indicated by an arrow) in TCGA GC expression profiles. Yellow and red represent high and low signature levels, respectively. The number of genes in the signatures are shown in parenthesis. **b** Kaplan–Meier survival curves are shown for TCGA GC patients with high and low CD133 expression signature levels (red and blue, respectively; left). Significance was estimated using the log-rank test and shown in the panel. Similarly analyzed for the level of stromal signatures (right) (**c**) For 21 tumor types including GC (STAD), hazard ratios for CIS and stromal signature levels (green and red lines, respectively) estimated by multivariate Cox regression are shown. Significance levels are indicated by asterisks with individual plots

To reduce the number of genes in the CD133 signatures for potential clinical utility, we selected 36 genes that appeared at least two times in three CD133/stemness-related signatures (“CD133-up”, “CD133-up-GBM” and “ESC1”). Of interest, PROM1 is the only gene commonly appeared in the three signatures. We annotate the 36 gene-sized signature as ‘core-in vitro-stemness’ (CIS) signature. The list of 36 CIS signature genes is available in Additional file 2: Table S5. CIS signature levels (asterisk in Fig. 5a) are correlated with those representing CD133-up and ESC, but inversely correlated with those of immune and stromal signatures in the expression of stomach cancers. This relationship among the signatures including CIS signature are consistently observed across 20 major tumor types as obtained from TCGA consortium (Additional file 1: Figure S6).

It was noted that the clinicopathological features associated with high CD133 expression signatures were associated with good prognostic GC features, such as intestinal type and low tumor GCs. in addition, high mutation burdens and MSI genotypes have also been considered good prognostic markers in GC [22], especially as potential predictive markers for immune checkpoint blockade treatment [25]. Thus, we first evaluated whether CIS signature levels are associated with clinical outcomes in GC patients. We observed that high and low CIS signature levels were associated with favorable and unfavorable patient overall survival rates, respectively (Fig. 5b; Log-rank *P* = 0.0461). The expression signature levels of stromal cells, which were inversely correlated with CD133 expression signatures, showed contrasting relationships with overall survival, i.e., the high and low stromal signature levels were associated with unfavorable and favorable patient survival, respectively (Fig. 5b; Log-rank *P* = 0.0032). This univariate analysis suggests that the CD133/stemness and stromal signature activity are favorable and unfavorable outcome predictors of GC, respectively. The relationship of patient survival with the stromal signature level is reasonable since the stromal signature levels may represent the extent of epithelial-to-mesenchymal transition, which is known as a poor prognostic factor of GC [22]. However, CD133 levels have been also proposed as unfavorable prognostic factors in previous reports [5–12], which is in contrast with our observation. We assume that this paradoxical result may come from the inverse relationship between the CD133 and stromal signature activities and we performed multivariate analysis taking two features into accounts simultaneously. Results of multivariate survival analysis of CIS and stromal signature levels are shown across 21 tumor types including GC (Fig. 5c). For the GC as well as BLCA and GBM, only the stromal signatures were associated with unfavorable survival while the CIS signature levels may not be significantly associated with patient survival after adjusting for the stromal effects. For seven tumor types (KIRC, LIHC, LUAD, PAAD, PRAD, SARC, and SKCM; see Methods for the abbreviations of tumor types), only the CIS signature levels showed significant relationship with patient survival as poor prognostic factors. In KIRP and LGG, both CIS and stromal signature activities were associated with unfavorable prognosis. These results suggests that the impact of CD133 and stromal activity are both unfavorable prognostic factors but the extent may be variable across tumor types.

## Discussion

In this study, we identified in vitro CD133 expression signatures from three GC cell lines and evaluated their expression-based signature levels and clinicopathological associations in primary GC datasets. Because of known GC heterogeneity [13] along with the substantial fraction of non-tumor cell components in primary tumors [19, 26], we chose cell lines to robustly detect the gene expression signatures associated with the activity of CD133 stem cell marker. We also obtained expression profiles of primary GCs (*n* = 18) with IHC-based CD133 positivity calls. A prediction test based on kNN-LOOCV showed that the prediction accuracy of CD133+ and CD133- were 100% and < 50% for cell lines and primary GC cases, respectively suggesting that the expression profiles of primary GCs may not discriminate the IHC-based CD133 positivity. This discrepancy may be due to the weak relationship between mRNA and protein abundance [27, 28]. In this study, we assume that one potential cause for the discrepancy between the IHC-based CD133 positivity and CD133 expression signature levels along with PROM1 expression may be the extent of stromal contamination. Since the epithelial tumor component is the major source for the CD133 signature activity with PROM1 expression, these features will be proportional to the tumor purity and also inversely correlated with stromal signature levels. It is controversial whether the tumor purity can be considered as intrinsic biological feature of tumors, but it is reasonable that tumor purity can be a confounding factor in genomic and clinical association studies [26, 29]. Because the IHC-based CD133 classification is commonly used, further investigation is needed to ascertain whether the transcriptome profiles of CD133+ and CD133- primary GCs are too heterogeneous to obtain robust signatures as well as to determine the extent to which the tumor purity or the stromal component impact the clinical evaluation of expression-based CD133 signatures.

For expression signature levels estimated from PanCancer-scale expression profiles including GC, the CD133 expression signatures were concordant with those of other tumors (GBM) and human ESCs. These transcriptional similarities across stem cell-related expression signatures emphasized the common molecular features associated with stem cells that are also present in primary cancers and stem cell lines. Additionally, the enriched molecular terms with CD133 signature genes and the correlations with the levels of other expression signatures, such as cell proliferation and cell cycle, suggested that cancer stem cells in GC and GBM might share a high level of cellular proliferation and reduced cell cycling [30]. This association has been interpreted as indicating that high CD133 expression signature levels are associated with more advanced tumors because somatic mutations accumulate during cancer progression [16]; however, the association of high CD133 expression levels in low tumor stages in our study suggested that the activity of CD133 may arise early in cancer development [31]. Moreover, the association of high CD133 expression signature with elevated mutation burdens in our study was largely attributed to the association of CD133 expression signature with MSI-H cancers. Given recent highlights on immune checkpoint blockade treatment in various cancer types including GC, the understanding for the association between the CD133 activity and hypermutation may be further required.

The IHC-based CD133 protein level quantification has been proposed as a prognostic marker for some tumors, including GC, but a clear relationship has not yet been established. In general, IHC-based CD133 positivity in GC has been regarded as a feature associated with high-stage and high-grade tumors with poor prognosis [5–12]. Our univariate analysis of CD133 signature (CIS signature) with patient survival showed that elevated CD133 activity may indicate favorable prognosis, which is in contrast with previous reports. First, we found that CD133 signature levels did not correlate with IHC-based CD133+/CD133- assignments suggesting that the protein level of the CD133 single-marker gene may not reflect the aggregated behavior of CD133-associated genes in the transcriptome. However, given the inverse correlation of CD133 signature activity with those of stromal cells, it is reasonable to assume that CD133 marker is specifically expressed in tumor cells and that the level of CD133 expression may be dependent on tumor purity in a similar manner as immune genes in tumor-infiltrating immune cells [29]. In the CD133 GBM study, the high levels of CD133 were associated with a GBM subtype with the highest tumor purity (i.e., GBM proneural group), and our findings that the CD133 expression signature levels were significantly correlated with tumor purity (Fig. 3g) may support this assumption. When we employed multivariate analysis considering two signature levels (CIS and stromal) simultaneously, we observed that both features represent unfavorable prognostic factors across multiple tumor types, but in a varying degree across tumor types. In GC genome, we assume that the stromal signature levels may reflect the extent of epithelial-to-mesenchymal transition, one of the known poor prognostic factors in GC genomes. Although CD133 activity is also an unfavorable prognostic factor, their inverse relationship with stromal signature score may lead to a discrepancy in the interpretation of their impact on patient survival. Thus, it requires a caution in evaluating the clinical impact of features that are associated with the tumor purity and stromal signature levels as we have observed for the impact of stromal signature on clinical outcomes dominates those of CD133 signature levels for at least three cancer types such as GC along with GBM and kidney cancers.

## Conclusions

Our findings indicated that the CD133 expression signature levels in GC cell lines showed transcriptional similarities with other stem cell-related expression signatures but were inversely correlated with those of tumor infiltrating stromal cells. The CD133 and stromal signature levels may be unfavorable prognostic factors across multiple cancer types including GC but their inverse relationship may influence their impact on clinical outcome.

## Additional files

Additional file 1: **Figure S1.** Fluorescence-activated cell sorting of CD133 in gastric cancer cell lines. CD133- cells were collected in overlapping area (about 6%) between isotype control (nonspecific staining) and CD133 staining for there cell lines by flow cytometry. CD133+ boundaries of three cell lines were set (about 10%) by clear division with negative population. **Figure S2.**. RNA expression levels of up- and down-regulated genes. The relative concentrations of RNA for CDC2 (the most up-regulated in CD133+ cell lines) and ARG1 (the most done-regulated in CD133+ cell lines) genes were measured by quantitative reverse transcription polymerase chain reaction (qRT-PCR). In three gastric cancer cell lines, CDC2 expression was higher in the CD133+ cell lines than those of CD133-. ARG1 expression was low in the CD133- KATO-III and SNU216 cell lines, but was not in the SNU601. **Figure S3.** Deconvolution of CD133 signatures. The relative abundance (%; *y*-axis) of CD133+ and CD133- signatures (red and blue, respectively) estimated by CIBERSORT algorithm are shown for 3 cell lines (CD133+ and CD133- in replicates). For two cell lines (KATO-III and SNU216), exclusive enrichment of CD133+ and CD133- signatures in the corresponding sorted cells. **Figure S4.** CD133 expression signature associated with MSI status. (a) TCGA stomach cases are distinguished into MSI-H, MSI-L and MSS cases and shown for the CD133 expression signature levels (*y*-axis). (b) CD133 expression signature levels are shown for the cases with or without the *MLH1* promoter methylation as a major genomic event associated with sporadic MSI-H. **Figure S5.** CD133 expression signature associated with clinical features. In an independent cohort of 300 GC primary cases (GSE62254), the correlative analyses with CD133 signature levels were performed for (a) Lauren classification, (b) tumor stages, (c) molecular subtypes, and (4) MLH1-IHC positivity. **Figure S6.** Relationship of CD133/stem cell signatures across 20 tumor types. Heatmaps are shown as the clustering results of CD133 and related signatures. Similarly analyzed with main Fig. 5a and CIS signature is marked with an asterisk. Seven and four gene sets that were segregated into two splits of main Fig. 5a (red and green, respectively) were consistently observed as two splits across 20 additional tumor types. (PPTX 223 kb)

Additional file 2: **Table S1.** Differentially expressed genes in CD133 + −vs.-CD133- gastric cancer cell lines. A total of 177 and 129 up- and down-regulated genes (SNR > 1.0 and SNR < − 1.0, respectively) in CD133+ cells compared to CD133- cells are listed with gene symbol and SNR. Type indicates whether the genes are up- or down-regulated in CD133_ cells. Additional information including the RefSeq ID, chromosome and gene descriptions are also shown. **Table S2.** Primers sequence of reverse transcription polymerase chain reaction. Primers of up-regulated CDC2 gene and down-regulated ARG1 genes in CD133+ cells are listed. **Table S3.** GO categories enriched with CD133 signature genes. The GO terms substantially enriched (*P* < 0.01; Fisher’s exact test) are listed for their categories (whether enriched in CD133+ up- or down-regulated genes). The number of genes in GO terms are Gene Size and the overlapping CD133 signature genes are DEG. Significance from Fisher’s exact test is *P* value. **Table S4.** Correlation of CD133 signature and clinicopathological features in GC. A total of 34 features were evaluated with CD133 signature as available in TCGA consortium. The types of statistical tests, significance level and the classes used for the tests are listed. **Table S5.** CIS signature. 36 genes were selected as those appeared at least twice in three CD133/stemness-related signatures. (XLSX 45 kb)

## Abbreviations

BLCA: Bladder urothelial carcinoma
BRCA: Breast invasive carcinoma
CESC: Cervical and endocervical cancers
COADREAD: Colorectal adenocarcinoma
ESC: Embyonic stem cell
ESCA: Esophageal carcinoma
GBM: Glioblastoma multiforme
GC: Gastric cancer
GSEA: Gene set enrichment analysis
HNSC: Head and neck squamous cell carcinoma
IHC: Immunohistochemistry
KIRC: Kidney renal clear cell carcinoma
KIRP: Kidney renal papillary cell carcinoma
kNN: k-nearest neighbors
LGG: Lower grade glioma
LIHC: Liver hepatocellular carcinoma
LOOCV: Leave-one-out-cross-validation
LUAD: Lung adenoma carcinoma
LUSC: Lung squamous cell carcinoma
MAD: Median absolute deviation
MSI: Microsatellite instability
OV: Ovarian serous cystadenocarcinoma
PAAD: Pancreatic adenocarcinoma
PRAD: Prostate adenocarcinoma
SARC: Sarcoma
SKCM: Skin cutaneous melanoma
SNR: Signal-to-noise ratio
THCA: Thyroid carcinoma
UCEC: Uterine corpus endometrial carcinoma
