# Supplementary material for: Impact of in vitro driven expression signatures of CD133 stem cell marker and tumor stroma on clinical outcomes in gastric cancers
Source: BMC Cancer. 2019 Feb 4;19:119. doi: 10.1186/s12885-019-5332-y (PMC6360664; doi:10.1186/s12885-019-5332-y)
Supplement: Supplementary file 1 — Figure S1. Fluorescence-activated cell sorting of CD133 in gastric cancer cell lines. CD133- cells were collected in overlapping area (about 6%) between isotype control (nonspecific staining) and CD133 staining for there cell lines by flow cytometry. CD133+ boundaries of three cell lines were set (about 10%) by clear division with negative population. Figure S2.. RNA expression levels of up- and down-regulated genes. The relative concentrations of RNA for CDC2 (the most up-regulated in CD133+ cell lines) and ARG1 (the most done-regulated in CD133+ cell lines) genes were measured by quantitative reverse transcription polymerase chain reaction (qRT-PCR). In three gastric cancer cell lines, CDC2 expression was higher in the CD133+ cell lines than those of CD133-. ARG1 expression was low in the CD133- KATO-III and SNU216 cell lines, but was not in the SNU601. Figure S3. Deconvolution of CD133 signatures. The relative abundance (%; y-axis) of CD133+ and CD133- signatures (red and blue, respectively) estimated by CIBERSORT algorithm are shown for 3 cell lines (CD133+ and CD133- in replicates). For two cell lines (KATO-III and SNU216), exclusive enrichment of CD133+ and CD133- signatures in the corresponding sorted cells. Figure S4. CD133 expression signature associated with MSI status. (a) TCGA stomach cases are distinguished into MSI-H, MSI-L and MSS cases and shown for the CD133 expression signature levels (y-axis). (b) CD133 expression signature levels are shown for the cases with or without the MLH1 promoter methylation as a major genomic event associated with sporadic MSI-H. Figure S5. CD133 expression signature associated with clinical features. In an independent cohort of 300 GC primary cases (GSE62254), the correlative analyses with CD133 signature levels were performed for (a) Lauren classification, (b) tumor stages, (c) molecular subtypes, and (4) MLH1-IHC positivity. Figure S6. Relationship of CD133/stem cell signatures across 20 tumor types. Heatmaps are shown [file 12885_2019_5332_MOESM1_ESM.pptx]

## Slide 1
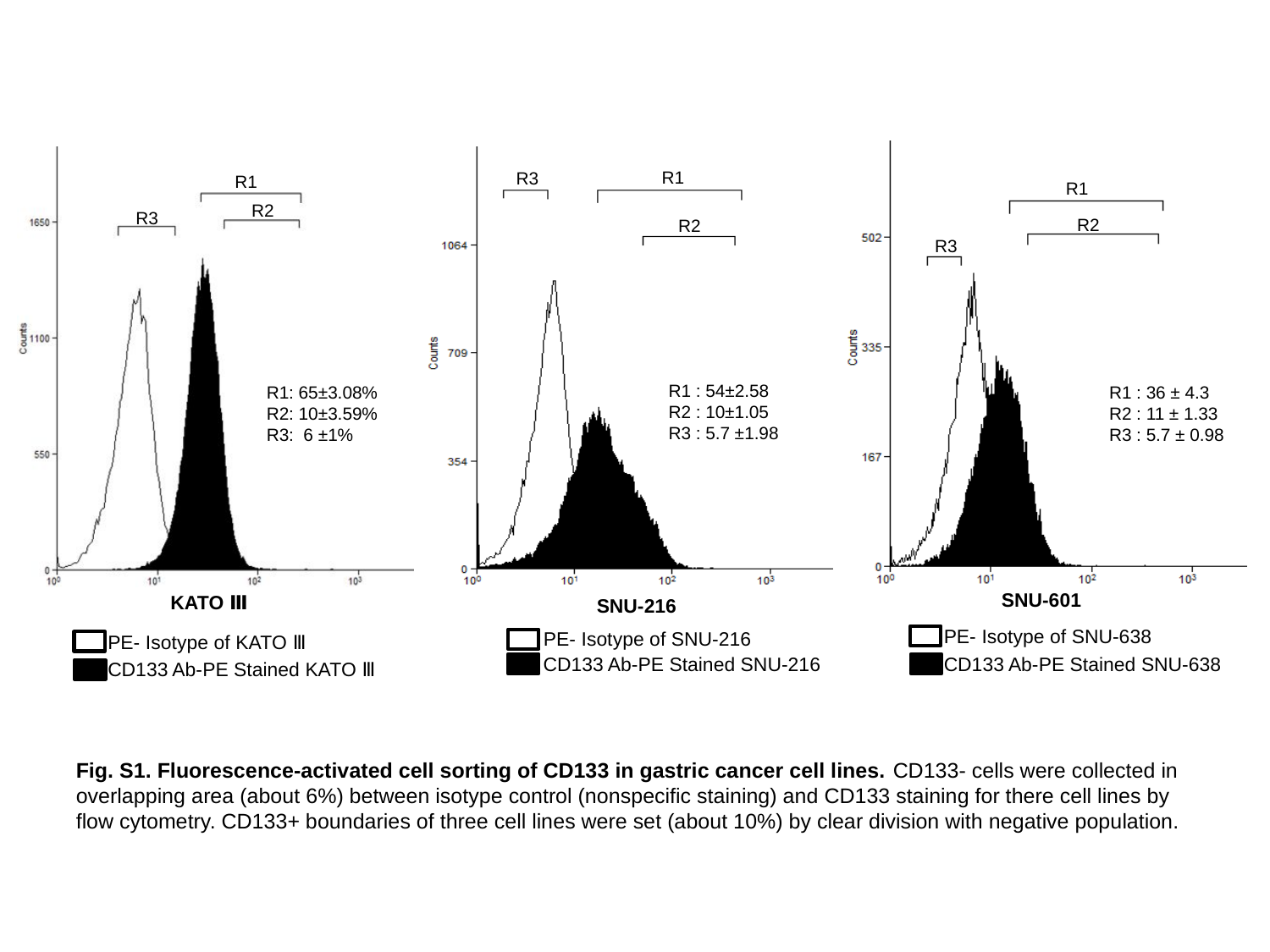

R1
R3
R1
R1
R2
R3
R2
R2
R3
R1 : 54±2.58
R2 : 10±1.05
R3 : 5.7 ±1.98
R1: 65±3.08%
R2: 10±3.59%
R3: 6 ±1%
R1 : 36 ± 4.3
R2 : 11 ± 1.33
R3 : 5.7 ± 0.98
SNU-601
KATO Ⅲ
SNU-216
PE- Isotype of SNU-638
CD133 Ab-PE Stained SNU-638
PE- Isotype of SNU-216
CD133 Ab-PE Stained SNU-216
PE- Isotype of KATO Ⅲ
CD133 Ab-PE Stained KATO Ⅲ
Fig. S1. Fluorescence-activated cell sorting of CD133 in gastric cancer cell lines. CD133- cells were collected in overlapping area (about 6%) between isotype control (nonspecific staining) and CD133 staining for there cell lines by flow cytometry. CD133+ boundaries of three cell lines were set (about 10%) by clear division with negative population.

## Slide 2
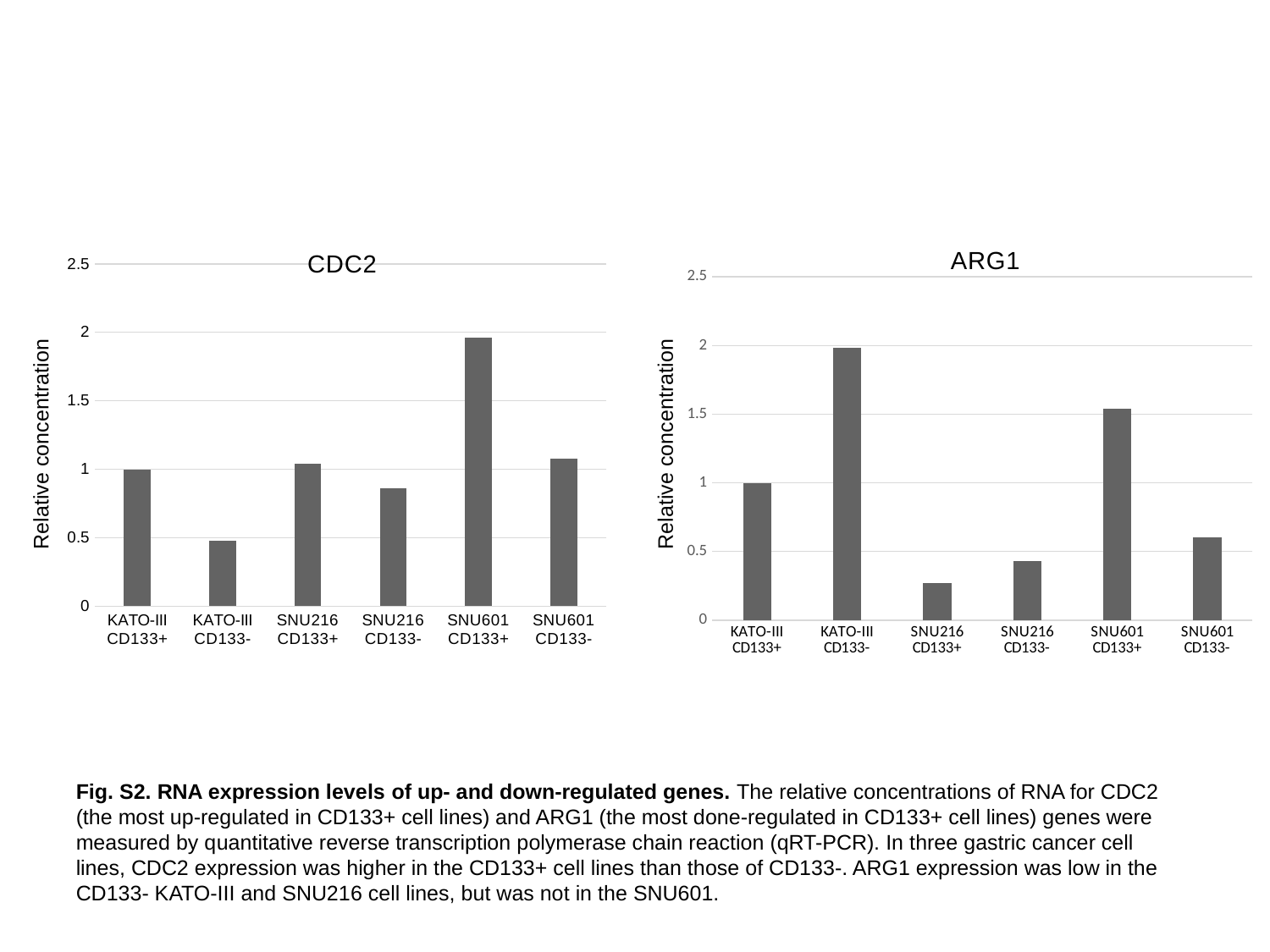

### Chart: ARG1
| Category | |
|---|---|
| KATO-III CD133+ | 1.0 |
| KATO-III CD133- | 1.9848580293051135 |
| SNU216 CD133+ | 0.2727365555415433 |
| SNU216 CD133- | 0.431705873482573 |
| SNU601 CD133+ | 1.5403119341888516 |
| SNU601 CD133- | 0.6013253421676722 |
### Chart: CDC2
| Category | |
|---|---|
| KATO-III CD133+ | 1.0 |
| KATO-III CD133- | 0.4762620058714614 |
| SNU216 CD133+ | 1.0426321253513 |
| SNU216 CD133- | 0.8608316192737728 |
| SNU601 CD133+ | 1.9629381081545456 |
| SNU601 CD133- | 1.0783586875932165 |Relative concentration
Relative concentration
Fig. S2. RNA expression levels of up- and down-regulated genes. The relative concentrations of RNA for CDC2 (the most up-regulated in CD133+ cell lines) and ARG1 (the most done-regulated in CD133+ cell lines) genes were measured by quantitative reverse transcription polymerase chain reaction (qRT-PCR). In three gastric cancer cell lines, CDC2 expression was higher in the CD133+ cell lines than those of CD133-. ARG1 expression was low in the CD133- KATO-III and SNU216 cell lines, but was not in the SNU601.

## Slide 3
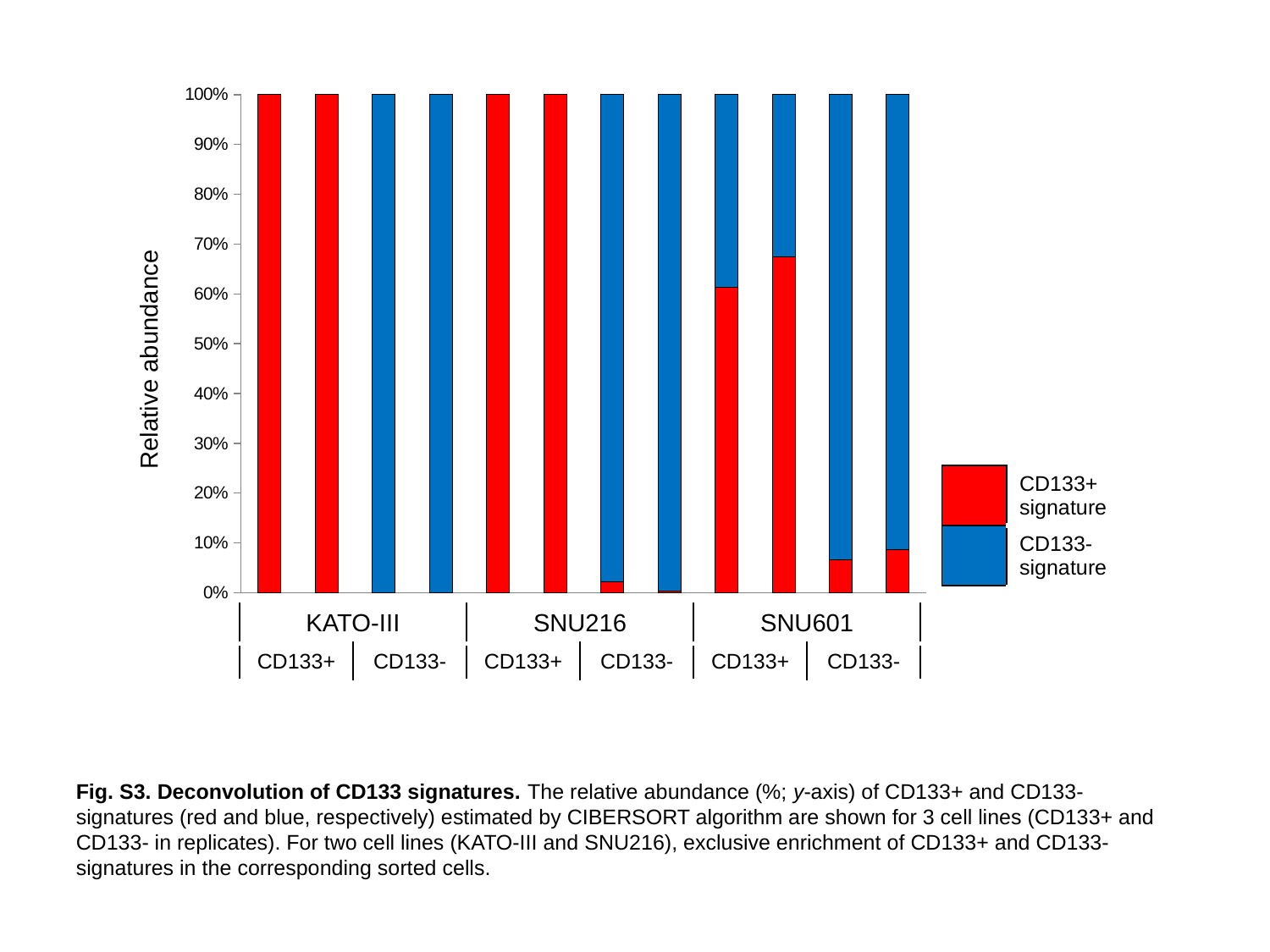

### Chart
| Category | CD133+ | CD133- |
|---|---|---|
| KATO-III-CD133+1 | 1.0 | 0.0 |
| KATO-III-CD133+2 | 1.0 | 0.0 |
| KATO-III-CD133-1 | 0.0 | 1.0 |
| KATO-III-CD133-2 | 0.0 | 1.0 |
| SNU-216-CD133+1 | 1.0 | 0.0 |
| SNU-216-CD133+2 | 1.0 | 0.0 |
| SNU-216-CD133-1 | 0.02219850439972798 | 0.9778014956002707 |
| SNU-216-CD133-2 | 0.0036964089589756046 | 0.9963035910410187 |
| SNU-601-CD133+1 | 0.61274332800227 | 0.387256671997731 |
| SNU-601-CD133+2 | 0.6752551007589 | 0.3247448992411005 |
| SNU-601-CD133-1 | 0.06685424224542803 | 0.9331457577545716 |
| SNU-601-CD133-2 | 0.08570183865043418 | 0.91429816134957 |Relative abundance
| | CD133+ signature |
| --- | --- |
| | CD133- signature |
| KATO-III | | SNU216 | | SNU601 | |
| --- | --- | --- | --- | --- | --- |
| CD133+ | CD133- | CD133+ | CD133- | CD133+ | CD133- |
Fig. S3. Deconvolution of CD133 signatures. The relative abundance (%; y-axis) of CD133+ and CD133- signatures (red and blue, respectively) estimated by CIBERSORT algorithm are shown for 3 cell lines (CD133+ and CD133- in replicates). For two cell lines (KATO-III and SNU216), exclusive enrichment of CD133+ and CD133- signatures in the corresponding sorted cells.

## Slide 4
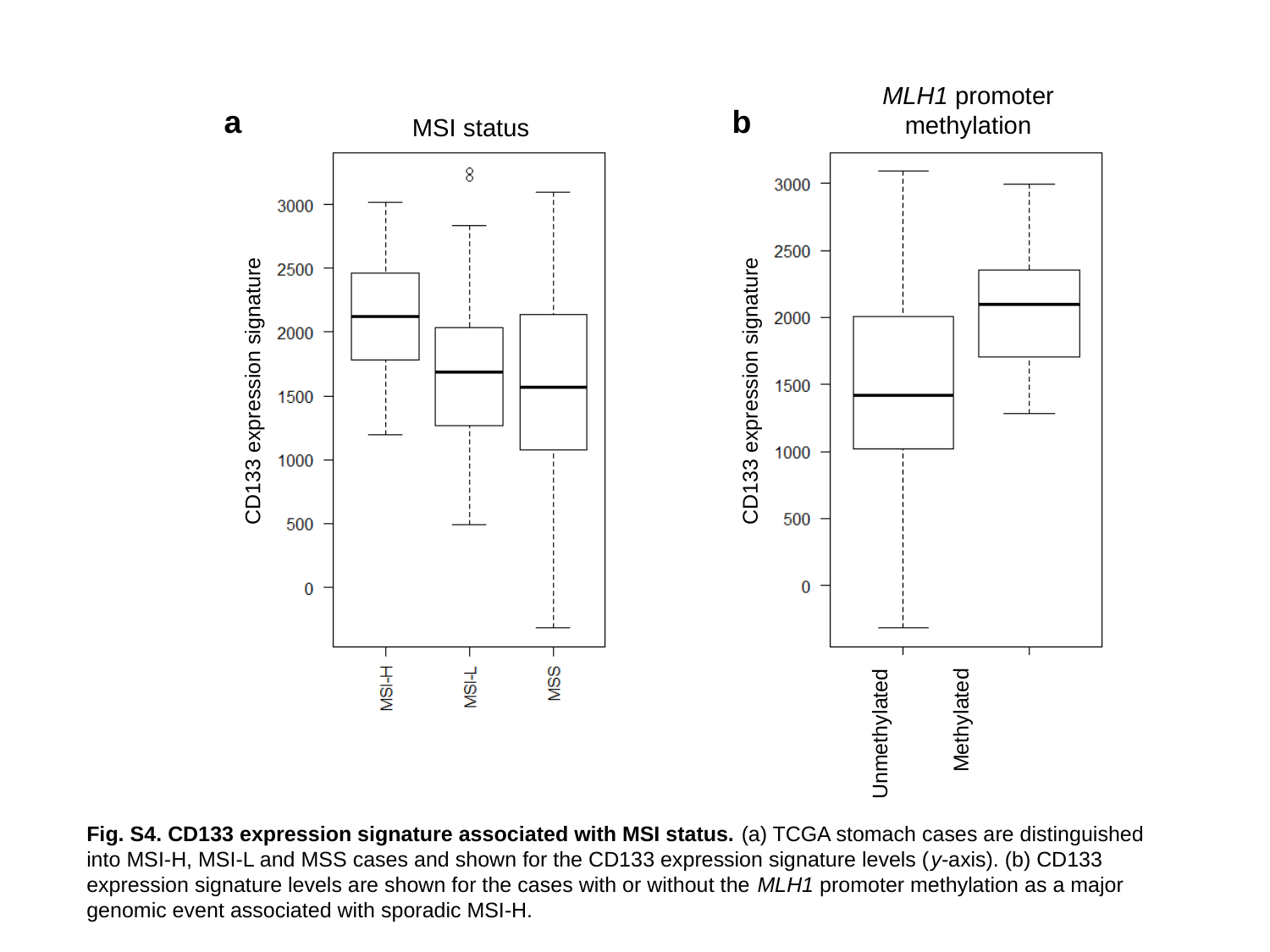

MLH1 promoter methylation
a
b
MSI status
CD133 expression signature
CD133 expression signature
| Unmethylated | Methylated |
| --- | --- |
Fig. S4. CD133 expression signature associated with MSI status. (a) TCGA stomach cases are distinguished into MSI-H, MSI-L and MSS cases and shown for the CD133 expression signature levels (y-axis). (b) CD133 expression signature levels are shown for the cases with or without the MLH1 promoter methylation as a major genomic event associated with sporadic MSI-H.

## Slide 5
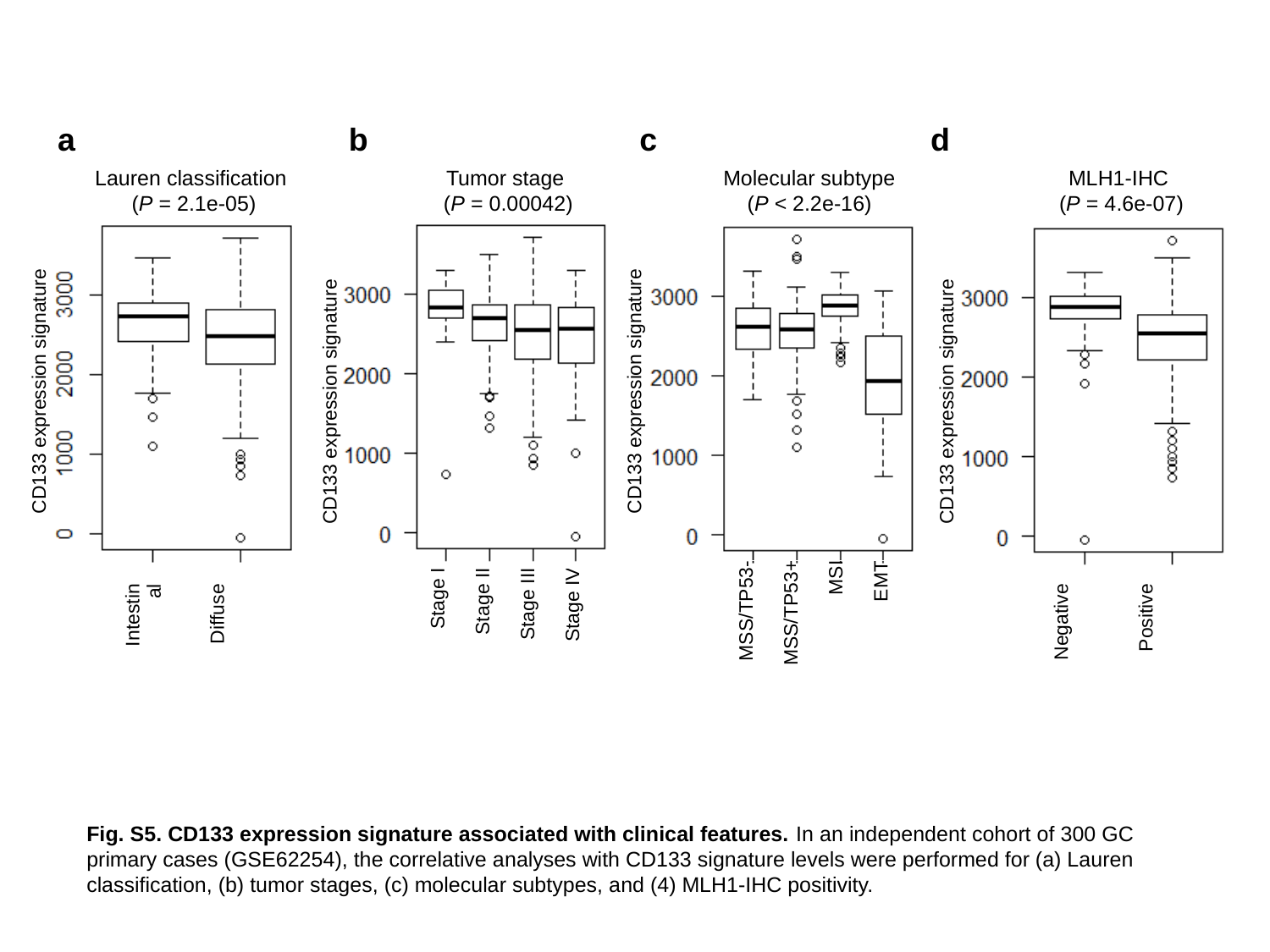

| a | b | c | d |
| --- | --- | --- | --- |
Lauren classification
(P = 2.1e-05)
Tumor stage
(P = 0.00042)
Molecular subtype
(P < 2.2e-16)
MLH1-IHC
(P = 4.6e-07)
CD133 expression signature
CD133 expression signature
CD133 expression signature
CD133 expression signature
| MSS/TP53- | MSS/TP53+ | MSI | EMT |
| --- | --- | --- | --- |
| Stage I | Stage II | Stage III | Stage IV |
| --- | --- | --- | --- |
| Intestinal | Diffuse |
| --- | --- |
| Negative | Positive |
| --- | --- |
Fig. S5. CD133 expression signature associated with clinical features. In an independent cohort of 300 GC primary cases (GSE62254), the correlative analyses with CD133 signature levels were performed for (a) Lauren classification, (b) tumor stages, (c) molecular subtypes, and (4) MLH1-IHC positivity.

## Slide 6
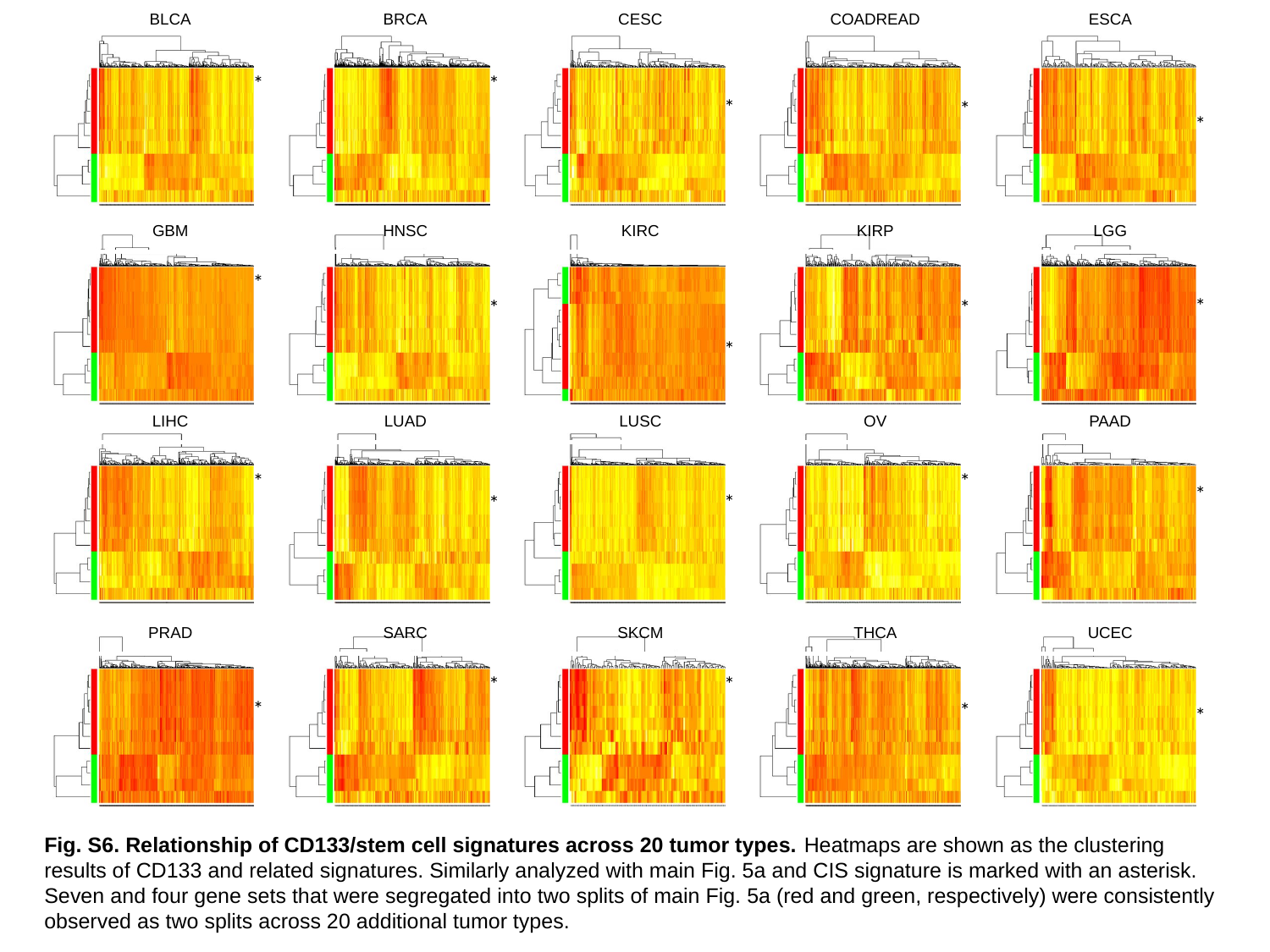

| BLCA | BRCA | CESC | COADREAD | ESCA |
| --- | --- | --- | --- | --- |
*
*
*
*
*
| GBM | HNSC | KIRC | KIRP | LGG |
| --- | --- | --- | --- | --- |
*
*
*
*
*
| LIHC | LUAD | LUSC | OV | PAAD |
| --- | --- | --- | --- | --- |
*
*
*
*
*
| PRAD | SARC | SKCM | THCA | UCEC |
| --- | --- | --- | --- | --- |
*
*
*
*
*
Fig. S6. Relationship of CD133/stem cell signatures across 20 tumor types. Heatmaps are shown as the clustering results of CD133 and related signatures. Similarly analyzed with main Fig. 5a and CIS signature is marked with an asterisk. Seven and four gene sets that were segregated into two splits of main Fig. 5a (red and green, respectively) were consistently observed as two splits across 20 additional tumor types.
